# Effect of a tea tree oil mouthwash on plaque-induced gingivitis when comparing to chlorhexidine: an exploratory randomised clinical trial

**DOI:** 10.3389/froh.2026.1727735

**Published:** 2026-06-03

**Authors:** Alfred Menat, Cecília Rozan, Helena Barroso, Luís Proença, Madalena Salema-Oom, João Botelho, Abdesselam Zhiri, Ana Cristina Manso

**Affiliations:** 1 Egas Moniz Center for Interdisciplinary Research (CiiEM), Egas Moniz School of Health & Science, Almada, Portugal; 2 Inula (Pranarôm), Ghislenghien, Belgium

**Keywords:** chlorhexidine, gingivitis, periodontal disease, preventive dentistry, streptococcus mutans, tea tree

## Abstract

Gingivitis, the most common periodontal disease, is an inflammatory condition of the gingiva resulting from bacterial biofilm accumulation without periodontal attachment loss. Chlorhexidine (CHX) is the gold standard for chemical plaque control, yet its prolonged use is associated with undesirable side effects. Tea tree essential oil (TTO, *Melaleuca alternifolia*), with documented antimicrobial and anti-inflammatory properties, has emerged as a potential natural alternative. This exploratory randomized, controlled, triple-blind clinical trial evaluated the antimicrobial, cytotoxic, and clinical effects of a 1% TTO-based mouthwash compared with 0.12% CHX in 30 adults with plaque-induced gingivitis. Both mouthwashes significantly reduced plaque and gingival indices as well as bacterial counts after 15 days (*p* < 0.001), with no significant intergroup differences in clinical outcomes. CHX achieved a greater reduction in bacterial load, while TTO presented fewer adverse effects, limited to mild taste alteration and transient increase in salivary flow. Both formulations exhibited *in vitro* cytotoxicity, though clinical tolerance was high. These results suggest that, under the tested conditions, TTO formulation at 1% (v/v) exerts an antibacterial effect comparable to CHX, while displaying a dose-dependent pattern.

**Clinical Trial Registration:**

https://doi.org/10.17605/OSF.IO/B5HCD.

## 1 Introduction

Dental biofilm-induced gingivitis is the most prevalent periodontal disease. It represents an inflammatory condition confined to the free and attached gingiva in response to the accumulation of bacterial biofilm at or immediately below the gingival margin, without periodontal attachment loss, and is reversible upon reduction of dental biofilm levels (1, 2). Clinically, gingivitis may present with oedema, increased sensitivity, erythema, swelling, and bleeding on probing at more than 10% of sites (over 30% in the case of generalised gingivitis) (1). Its multifactorial nature accounts for the wide variation in prevalence depending on the population studied. In Portugal, an average prevalence of 26.9% has been reported in the adult population, with higher rates among women aged 31–40 years, and reaching 88% in pregnant women (3, 4).

Management primarily relies on rigorous mechanical plaque control, both self-administered and professional, reinforced by professional oral hygiene instructions (5). Adjunctive use of chemical plaque control agents, such as mouthrinses, appears to enhance the reduction of gingival inflammation and plaque accumulation compared with the use of toothpaste alone (6). If left untreated, gingivitis may progress to periodontitis, an irreversible stage characterised by the destruction of periodontal support, influenced by biofilm composition, host inflammatory response, genetic and environmental factors, and poor oral hygiene (7–9). Early diagnosis and treatment are therefore essential, as periodontitis has been associated with several systemic complications, including an increased risk of stroke, myocardial infarction, Alzheimer's disease, and cardiovascular disorders (10, 11).

*Streptococcus mutans* first isolated in 1924 by J. K. Clarke (12) plays a pivotal role in biofilm development. This bacterium expresses surface glucosyltransferases (GTFs) that metabolise dietary sucrose into glucans, which constitute extracellular polysaccharides (EPS) of the matrix and act as binding receptors for other colonising bacteria (13). Although it is not directly linked to an increased prevalence of gingivitis, its role in biofilm formation indirectly favours gingival inflammation (8, 14). Moreover, dissemination of *S. mutans* from the oral cavity into the bloodstream has been implicated in systemic diseases, including cerebral haemorrhage, tumour metastasis, atherosclerosis, infective endocarditis, non-alcoholic steatohepatitis (NASH), Immunoglobulin A nephropathy (IgAN), and inflammatory bowel disease (IBD) (15, 16).

Chlorhexidine digluconate (CHX), developed in the 1940s, is a broad-spectrum antimicrobial effective against viruses, fungi, yeasts, and both Gram-positive and Gram-negative bacteria, including *S. mutans*, on which it exerts prolonged inhibitory effects (17, 18). Its action involves passive absorption into the microbial membrane, leading to altered permeability, enzyme inhibition, and leakage of cytoplasmic components (19). In the oral cavity, CHX binds to anionic surfaces such as mucosa, teeth, and salivary glycoproteins of the enamel-acquired pellicle, preventing microbial adhesion and conferring notable substantivity (up to 5 h in saliva and 12 h on mucosal surfaces). CHX is widely recognised as the gold standard for gingivitis management and biofilm control, owing to its spectrum of activity and oral substantivity (19). However, long-term use is associated with adverse effects—including dental and mucosal staining, dysgeusia, increased calculus formation, mucosal irritation, black hairy tongue, and parotid swelling (17, 20–22) —and may contribute to antibiotic resistance, although further evidence is required (23, 24).

Essential oils (EOs) have long been employed in dentistry, from 19th-century endodontics to the treatment of oral pathologies in the 1990s, mainly due to their antibacterial activity unaffected by antibiotic resistance (25, 26). Tea tree oil (TTO), obtained from *Melaleuca alternifolia*, comprises over one hundred constituents, of which terpinen-4-ol, 1,8-cineole and α-terpineol are the most relevant, displaying broad antimicrobial activity against bacteria, fungi, viruses and protozoa (26–28). Their synergistic action enhances efficacy compared with isolated components (29). These molecules, owing to their small size and low molecular weight, rapidly penetrate microbial membranes, disrupting their structure, inducing leakage of intracellular contents and causing osmotic imbalance (27, 28, 30, 31). TTO also impairs peptidoglycan synthesis by inactivating glycosyltransferase, compromising bacterial cell walls (27). In addition, terpinen-4-ol demonstrates anti-inflammatory effects by inhibiting pro-inflammatory cytokines (IL-6, TNF-α) and modulating signalling pathways such as NF-*κ*B and mTOR (32, 33). Toxic effects are rare, mostly associated with accidental ingestion of concentrated TTO, which may induce central nervous system depression but has never been fatal (27, 34). Inadequate storage may increase oxidation and allergenicity (35). Its use remains contraindicated in pregnancy and lactation (36, 37).

Beyond its general antimicrobial effects, TTO has demonstrated efficacy in oral candidiasis, chronic gingivitis and HSV-1 lesions, as well as in resistant infections such as Methicillin-resistant *Staphylococcus aureus* (MRSA), Vancomycin-resistant *Enterococcus* (VRE) and Extended-spectrum *β*-lactamase (ESBL)–producing *Escherichia coli* (27, 29, 34, 38). Synergistic effects with antibiotics have been observed, and a TTO-based body wash proved more effective than Chlorhexidine in MRSA decolonisation in children (39). In periodontology, adjunctive subgingival 5% TTO gel has been associated with reductions in probing depth and gains in clinical attachment at 3–6 months vs. scaling alone (40). Preclinical evidence further suggests pro-apoptotic effects in melanoma and oral squamous cell carcinoma cells without mutagenic activity (41, 42). Liposomal formulations may improve stability, bioactivity and tolerability, supporting exploration of TTO within biomaterials (43).

This exploratory randomized, controlled, triple-blind clinical trial evaluated the antimicrobial, cytotoxic, and clinical effects of a 1% TTO-based mouthwash compared with 0.12% CHX in adults with plaque-induced gingivitis.

## 2 Material and methods

The study protocol was prospectively registered on the 06/10/2025 in the OSF Registries under the identifier https://doi.org/10.17605/OSF.IO/B5HCD. This manuscript is reported in accordance with the CONSORT 2025 Statement (44) and its Checklist included as Supplementary Material.

### 2.1 Ethical considerations, randomization and blinding

The study protocol was reviewed and approved by the Ethics Committee of Egas Moniz School of Health & Science (protocol code PT-316/24/1561 on February 2025). All participants were informed about the study objectives, procedures, and potential risks before enrolment, and provided written informed consent prior to participation. Data collection and handling followed strict confidentiality standards, ensuring full anonymization of participant information. All experimental procedures complied with the ethical principles of the Declaration of Helsinki and with institutional regulations governing research involving human participants.

Participants were randomly allocated to the study groups using a computer-generated randomization sequence. Allocation concealment was ensured using sequentially numbered, opaque, sealed envelopes prepared by an independent researcher not involved in recruitment, intervention delivery, or outcome assessment.

The patients, the clinical examiner, and the microbiological analyst were blinded to group allocation. Patient blinding was ensured through the use of identical vials for delivery of the study preparations, which were indistinguishable in appearance. The group allocation code was only disclosed after completion of data collection and statistical analysis.

### 2.2 Formulation and preparation of the 1% tea tree oil mouthwash

The mouthwash was prepared under aseptic conditions to ensure stability and reproducibility of the formulation. The solubilisation of TTO in water was achieved using the non-ionic surfactant polysorbate 80 (Tween-80, Sigma-Aldrich). Equal volumes of TTO and Tween-80 (3 mL each) were measured separately and mixed in a sterile, inert, dark-glass bottle to prevent light-induced degradation and chemical interaction with the container. The final volume was adjusted to 300 mL with sterile demineralised water to achieve a final TTO concentration of 1% (v/v). The mouthwash bottles were subsequently coded according to the identification number of the participant to whom they were assigned.

### 2.3 Assessment of antimicrobial activity and biocompatibility

#### 2.3.1 Antimicrobial activity in liquid Medium

The antimicrobial activity of Tea Tree Oil (TTO) was evaluated using a liquid-phase assay against *Streptococcus mutans* ATCC 55676. A bacterial suspension in Brain Heart Infusion (BHI) broth, adjusted to 1 on the McFarland scale, was distributed into a 48-well microplate and exposed in triplicate to different test formulations (1:1 ratio, 200 µL each): Chlorhexidine (CHX, 0.12%) as a positive control, TTO-based formulations at 0.25%, 0.5%, and 1% (v/v), pure TTO (100%), and an aqueous Tween-80 solution (1% v/v) as a negative control. A growth control (bacterial suspension + BHI) and a sterility control (BHI only) were also included.

After incubation for 48 h at 37°C under continuous shaking (70 RPM), the optical density (OD) at 600 nm was measured using a TECAN® Infinite M Plex microplate reader following 10 s of agitation. The bacterial growth inhibition rate was then calculated using the following equation (26):

$$Inhibition\;rate=\frac{(ODcontrol-ODtreatment)*100}{\left(ODcontrol\right)}$$

#### 2.3.2 Cytotoxicity assessment

The biocompatibility of the mouthwash was evaluated using a cytotoxicity assay with a mouse embryonic fibroblast cell line (NIH/3T3; Sigma-Aldrich) in accordance with ISO 10993-5:2009 (45). The cells were seeded in 96-well plates, in quintuplicate, at a density of 1 × 10^4^ cells per well in Dulbecco's Modified Eagle's Medium (DMEM) supplemented with 10% foetal bovine serum and 1% antibiotics and incubated at 37°C with 5% CO_2_ for 24 h. After 24 h of exposure to serial dilutions (1:2, 1:4, and 1:8) of either the TTO-based mouthwash (1% v/v stock solution containing 1% Tween 80) or the CHX (0.12% v/v stock solution Bexident®) in supplemented DMEM, cell viability was determined using the MTT assay. Untreated control exposed to DMEM only and a control exposed to 1% (v/v) Tween 80 were also considered.

Absorbance was measured at 565 nm, and cell viability was expressed relative to the untreated control, which was considered to be 100% viability (46).

$$Cell\;viability\;(\text{\%})=\left(\frac{Absobance\;treatment}{Absorbance\;negative\;control}\right)*100$$

### 2.4 Clinical protocol

#### 2.4.1 Participants and eligibility criteria

Thirty-five participants were recruited from the Egas Moniz University Clinic (CUEM). All signed informed consent prior to participation and met the study's inclusion criteria. This sample was set considering the material and conditions available to ensure the feasibility of the study.

Participants were eligible for inclusion if they were aged 18 years or older, presented with at least 20 natural teeth, exhibited clinical signs of gingival inflammation, and provided written informed consent. Exclusion criteria comprised individuals younger than 18 years, those with active periodontal disease or probing depth greater than 4 mm, pregnant or breastfeeding women, users of fixed orthodontic appliances, and individuals with systemic diseases or known allergies to any mouthwash component. Participants who had taken antibiotics, corticosteroids, or antiseptics within the previous three months were also excluded. Smoking habits and the use of medications associated with gingival enlargement were recorded but not considered exclusion criteria, as these factors were not expected to influence intra-individual comparisons between baseline (T0) and post-treatment (T1) outcomes.

### 2.5 Clinical protocol

Participants were randomly allocated into two groups using a computer-generated randomisation sequence: a control group using a Chlorhexidine-based mouthwash (CHX, 0.12%) and an experimental group using a Tea Tree Oil-based mouthwash (TTO, 1%). At baseline (T0), all participants completed a structured questionnaire covering sociodemographic and clinical background, oral hygiene habits, and self-perception of gingival health. Clinical procedures included photographic recording, saliva collection, assessment of the Gingival Index (GI) as per Loe & Silness, and Plaque Index (PI), scaling, and motivation for oral hygiene. Saliva samples were subjected to microbiological analysis for quantification of the oral bacterial flora.

Each participant received an anonymised, identical bottle containing the mouthwash assigned to their group. Both groups used their assigned mouthwash for 15 consecutive days following the same protocol. After this period (T1), the same clinical and microbiological procedures were repeated, together with a final questionnaire addressing participants' experience, perceived changes in gingival condition, and oral hygiene adherence. After initial enrolment, seven participants were excluded because they did not meet eligibility criteria, resulting in a final sample of 28 subjects (14 per group). Data obtained at T0 and T1 were statistically compared to assess changes in clinical indices and bacterial counts within and between groups.

### 2.5 Saliva samples collections and microbiological analysis

For microbiological identification and quantification of oral microorganisms, unstimulated saliva was collected at baseline (T0) and after 15 days (T1) using Salivette® Cortisol collection tubes (SARSTEDT®), following the manufacturer's instructions. Serial tenfold dilutions were prepared in sterile distilled water (up to 10^−5^), and 100 µL of each dilution was spread onto Columbia agar with 5% sheep blood (GS) and Mitis Salivarius Agar (MSA). For T0 samples, GS plates were inoculated with dilutions from 1:100 to 1:100 000, and MSA plates with 1:10 to 1:10 000; for T1 samples, GS plates received dilutions from 1:10 to 1:10 000, and MSA plates from 1:1 to 1:1 000. All plates were incubated at 37°C for 48 h under anaerobic conditions prior to colony quantification.

### 2.6 Statistical analysis

The initial data collection was recorded in Microsoft® Office Excel® files. All data were subjected to descriptive and inferential statistical analysis using IBM® SPSS® Statistics software (version 30.0). For inferential analysis, a significance level of 5% (*p* < 0.05) was established. The *p*-values were obtained using Student's *t*-test for paired samples (intragroup comparisons) and Student's *t*-test for independent samples (intergroup comparisons).

## 3 Results

### 3.1 Participants

**Figure 1:**
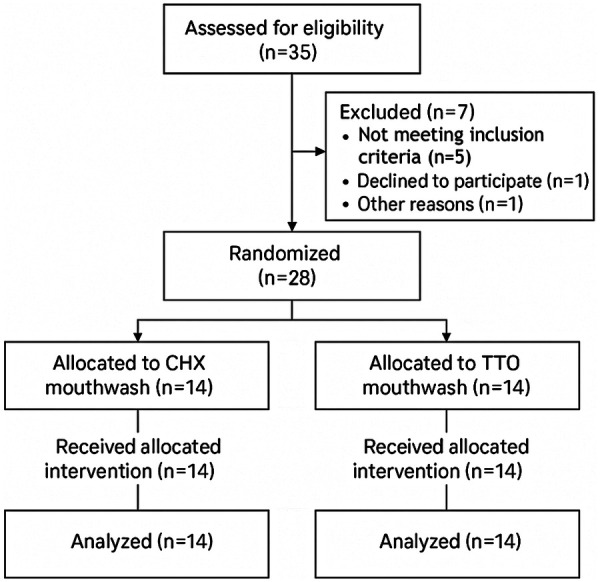
Patients flowchart.

A total of 35 individuals were assessed for eligibility (Figure 1). Seven did not meet the inclusion criteria or declined participation, resulting in a final randomized sample of 28 participants (14 per group). The study population comprised 60.7% females and 39.3% males, with a mean age of 32.5 ± 8.4 years. Most participants were non-smokers (64%) and reported brushing their teeth two to three times daily (96.4%). All participants completed the 15-day intervention and follow-up assessments, and no protocol deviations were recorded.

**Figure 2:**
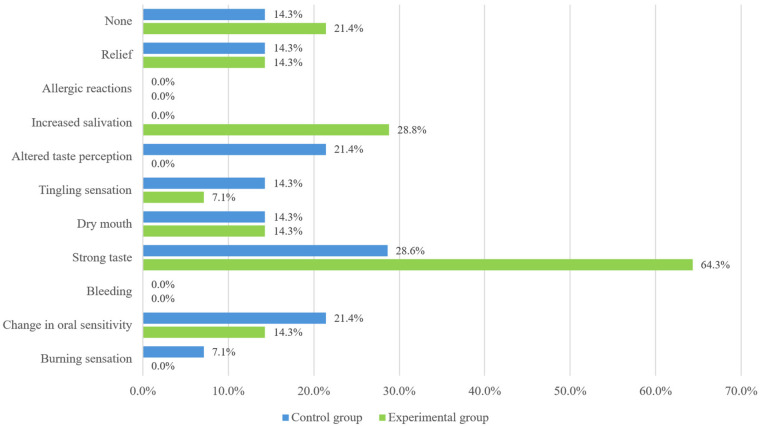
Side effects reported by patients in the control group and in the experimental group after the 15-day protocol.

### 3.2 *In vitro* results

#### 3.2.1 Antimicrobial activity in liquid medium

The liquid-phase assay revealed a significant inhibitory effect on *Streptococcus mutans* growth for both Chlorhexidine (CHX, 0.12%) and the TTO-based mouthwash at 1% (v/v). The inhibition rates were 72.75% ± 2.18% and 74.88% ± 3.84%, respectively, indicating a comparable antimicrobial efficacy between the two formulations. TTO showed a clear dose-dependent response, with inhibition rates of 61.58% ± 2.35% at 0.25% (v/v), 66.72% ± 2.11% at 0.5% (v/v), and 74.88% ± 3.84% at 1% (v/v).

However, since the TTO formulation contained Tween-80 (1% v/v), which itself exhibited an inhibition rate of 55.7% ± 3.15%, the antimicrobial activity observed cannot be attributed solely to the essential oil. The combined presence of TTO and the surfactant likely resulted in a synergistic effect that enhanced bacterial growth inhibition. These results suggest that, under the tested conditions, TTO at 1% (v/v) exerts an antibacterial effect comparable to CHX, while displaying a dose-dependent pattern.

#### 3.2.2 Cytotoxicity assessment

After 24 h of exposure, both the TTO (1% v/v) and the CHX (0.12% v/v) solutions induced a marked reduction in NIH/3T3 cell viability, with values around 1%. None of the tested dilutions (1:2, 1:4, or 1:8) of either formulation resulted in higher cell viability. Similarly, cells exposed to 1% Tween-80, the emulsifier used to prepare the TTO formulation, showed no viability.

### 3.3 Clinical results

Both mouthwashes produced significant improvements in clinical and microbiological parameters after 15 days of use (Table 1). In the TTO, mean PI decreased from 0.94 (±0.33) at T0 to 0.45 (±0.13) at T1, corresponding to a mean reduction of 0.48 units (*p* < 0.001) and a relative decrease of 51.1%. In the control group (CHX), PI decreased from 0.69 ± 0.37 to 0.19 ± 0.16, showing a mean reduction of 0.50 units (*p* < 0.001) and a 72.5% decrease. The intergroup comparison revealed no statistically significant difference (*p* = 0.889). Similarly, the Gingival Index (GI) improved significantly in both groups: from 0.54 ± 0.19 to 0.19 ± 0.07 in the TTO group (mean reduction 0.35; 64.8%; *p* < 0.001) and from 0.49 ± 0.13 to 0.18 ± 0.11 in the CHX group (mean reduction 0.31; 63.3%; *p* < 0.001), with no significant intergroup difference (*p* = 0.560).

**Table 1:** Comparison of clinical and microbiological outcomes after 15 days of mouthwash use.

Parameter	Group	MD	SD	Mean reduction (%)	Intragroup *p*	Intergroup *p*
PI	TTO	−0.48	0.32	51.1	<0.001	
	CHX	−0.50	0.27	72.5	<0.001	0.889
GI	TTO	−0.35	0.16	64.8	<0.001	
	CHX	−0.31	0.12	63.3	<0.001	0.560
*Streptococcaceae* spp. counts (Log CFU/mL)	TTO	−0.74	0.62	13.6	<0.001	
	CHX	−1.63	0.92	32.1	<0.001	0.006
Anaerobic microorganisms (Log CFU/mL)	TTO	−0.48	0.40	8.1	<0.001	
	CHX	−1.11	0.50	20.2	<0.001	<0.001

### 3.4 Microbiological results

Regarding microbiological outcomes (Table 1), *Streptococcus* spp. counts decreased from 5.45 ± 1.07 to 4.71 ± 1.16 Log(CFU/mL) in the TTO group (mean reduction 0.74; 13.6%; *p* < 0.001) and from 5.08 ± 1.17 to 3.45 ± 1.50 Log(CFU/mL) in the CHX group (mean reduction 1.63; 32.1%; *p* < 0.001). The intergroup comparison showed a significantly greater bacterial reduction in the CHX group (*p* = 0.006). Overall, both formulations achieved comparable clinical improvements, while CHX exhibited stronger antibacterial activity.

In the experimental group, the mean total anaerobic bacterial load (determined using GS) was 5.90 ± 0.83 Log(CFU/mL) at baseline (T0), decreasing to 5.42 ± 0.82 Log(CFU/mL) after treatment (T1), corresponding to a mean reduction of 0.48 logarithmic units (*p* < 0.001) and a relative decrease of 8.1%. In the control group, the mean value decreased from 5.50 ± 1.18 Log(CFU/mL) at baseline to 4.39 ± 1.22 Log(CFU/mL) at T1, representing a mean reduction of 1.11 logarithmic units (*p* < 0.001) and a 20.2% decrease. Statistical analysis revealed a significant difference in the reduction of total anaerobic microorganisms between groups (*p* < 0.001 < 0,05), indicating that the decrease in bacterial load was more pronounced in the control group than in the experimental group.

#### 3.4.1 Self-reported side effects

In the experimental group, the most frequently reported effect was a strong taste (64.3%), followed by increased salivation (28.6%). Other effects included changes in oral sensitivity (14.3%), dry mouth (14.3%), and a sensation of relief (14.3%).

In the control group, a strong taste was also the most common effect (28.6%), followed by altered taste (21.4%) and changes in oral sensitivity (21.4%). Reports of relief, dry mouth, tingling (14.3% each), and burning sensation (7.1%) were also recorded. Clinically, pigmentation of dental surfaces and black hairy tongue (events typically associated with prolonged CHX use) were observed in some participants from the control group.

## 4 Discussion

This randomized controlled trial compared the clinical and microbiological effects of a TTO-based mouthwash with those of CHX in individuals with plaque-induced gingivitis. Both interventions significantly reduced plaque accumulation and gingival inflammation after 15 days of use, confirming the short-term effectiveness of chemical plaque control adjuncts to mechanical hygiene. Despite slightly lower antimicrobial activity of TTO *in vitro*, clinical improvements in GI and PI were comparable to those achieved with CHX, suggesting that TTO may exert additional anti-inflammatory effects that contribute to gingival health improvement.

### 4.1 Interpretation and implications

The liquid-phase assay confirmed the significant antimicrobial activity of the 1% (v/v) TTO-based mouthwash, with inhibition rates comparable to Chlorhexidine (CHX, 0.12%), the gold standard for chemical plaque control (17). The effect was dose-dependent, increasing proportionally with TTO concentration. However, since the formulation contained Tween-80 (1% v/v), which exhibited its own antimicrobial effect, the observed activity likely reflects a synergistic interaction between the oil and the surfactant rather than the sole action of TTO. Further studies using alternative emulsifiers (such as lecithin-based emulsifiers, saponins, or food-grade biocompatible surfactants) or separated component testing are therefore required to determine the individual contribution of TTO to the overall antimicrobial effect.

After 24 h, both TTO and CHX had markedly reduced the viability of NIH/3T3 cells to around 1%, with the same results observed for dilutions of 1:2, 1:4 and 1:8. Notably, Tween 80 alone caused similar cytotoxicity, indicating that it may substantially contribute to the overall effect observed for the TTO formulation. These findings align with previous reports indicating low IC_50_ values for TTO (0.01–0.09% v/v) in fibroblasts and epithelial cell lines and confirm the well-documented cytotoxic of CHX across oral cell lines (32, 47, 48). The absence of viability at all TTO dilutions indicates a rapid cytotoxic response, independent of moderate concentration reductions, that is consistent with the membrane-disruptive mechanisms of TTO and CHX (19, 27). Nevertheless, these results should be interpreted with caution, as in clinical use oral tissues are exposed to mouthwashes for only a short time (approximately 30 s), followed by saliva dilution. Consequently, the 24 h exposure used *in vitro* likely overestimates cytotoxicity in real oral conditions. Future assays employing shorter incubation periods, as permitted by ISO standards (≥4 h), could better approximate clinically relevant exposure conditions.

While TTO shows promise as an antimicrobial agent, the high level of cytotoxicity observed *in vitro* raises concerns regarding biocompatibility in oral applications. Alternative formulations such as nanoencapsulation or the use of less toxic surfactants may enhance safety while preserve antimicrobial efficacy (43, 49). This study confirmed that a TTO-based mouthwash at 1% (v/v) can achieve clinical improvements in PI and GI comparable to those obtained with CHX, 0.12%. Although CHX showed greater bacterial reduction in the microbiological analysis, both formulations produced significant clinical improvements, suggesting that TTO's effects may extend beyond its antimicrobial capacity. The anti-inflammatory action of terpinen-4-ol, the major constituent of TTO, likely contributes to this outcome by modulating pro-inflammatory mediators such as IL-1β and TNF-α (32, 33). The results align with previous reports indicating that TTO can reduce gingival inflammation and bleeding while presenting fewer adverse effects than CHX (50, 51). The systematic review by Singh et al. (50) concluded that TTO-containing products can produce significant reductions in plaque and gingival indices, with efficacy in some studies approaching that of CHX. However, the authors also highlighted substantial heterogeneity among the included trials, particularly regarding TTO concentration, formulation, and duration of use, which limits direct comparability across studies. More recently, Mahapatra et al. (51) reported that a 0.2% TTO mouthwash led to significant improvements in clinical and microbiological parameters compared with placebo. Notably, the concentration used in that study was five times lower than the 1% (v/v) formulation tested in the present trial, yet still produced measurable clinical benefits. This suggests that TTO may exert biologically relevant effects even at relatively low concentrations, while also raising important questions regarding the optimal balance between efficacy and biocompatibility. The higher concentration employed in the current study may partly explain the magnitude of clinical improvement observed, but it also reinforces the need for further dose–response studies to establish the minimum effective concentration and to optimize safety profiles for routine clinical use. Despite its lower antibacterial potency, the comparable clinical benefits observed here reinforce the therapeutic potential of TTO as a safe and effective adjunct in plaque control.

Tolerability outcomes further support this perspective. While both mouthwashes caused transient local effects, CHX users experienced more frequent alterations in taste perception and oral sensitivity, as well as clinically visible pigmentation and black hairy tongue—well-documented effects of prolonged CHX use (19, 52). In contrast, no severe reactions occurred with TTO, and compliance remained high despite reports of an intense taste. Overall, these findings pave the way for larger trials to test the reliability of TTO as a promising alternative to CHX, capable of delivering comparable short-term clinical results with a more favourable safety profile. The cytotoxicity observed *in vitro* under prolonged exposure does not appear to reflect clinical intolerance during typical mouthwash use. Nonetheless, larger and longer-term studies are needed to confirm these results and to optimise TTO formulations for improved stability and biocompatibility.

### 4.2 Limitations

A limitation of the present study is that participants’ oral home-care products were not systematically assessed. The type of toothpaste used [particularly formulations containing desensitizing agents (e.g., potassium nitrate, arginine), stannous fluoride, or other active ingredients] may influence gingival inflammation and plaque accumulation independently of the tested mouthwashes (53). Likewise, the use of adjunctive products such as other mouthrinses (e.g., essential oil–based, CPC-containing, or herbal formulations) may modify biofilm composition and gingival outcomes, potentially acting as confounding factors. Although participants were instructed not to modify their usual oral hygiene habits during the study period, future trials should include a structured assessment of home-care tools and products, or implement standardized toothpaste and rinse protocols, to better control for these variables and strengthen internal validity.

Another important limitation of this study relates to the professional scaling and oral hygiene motivation provided before the delivery of the mouthwash bottles. This intervention alone is known to significantly reduce plaque levels and gingival inflammation and may therefore have contributed substantially to the clinical improvements observed in both groups, potentially attenuating the ability to detect differences attributable solely to the tested mouthwashes. However, this approach was intentionally adopted to standardize baseline oral conditions and to minimize inter-individual variability in plaque accumulation and hygiene practices, a strategy commonly used in clinical trials evaluating adjunctive chemical plaque control agents. Despite this standardization, the possibility remains that the magnitude of improvement observed reflects, at least in part, the effect of the initial professional intervention rather than exclusively the action of TTO or CHX. Future studies could address this limitation by incorporating longer wash-in periods, delayed randomization, or alternative study designs (e.g., no-initial-scaling control groups) to better isolate the specific effects of the tested formulations. As already stated, Tween-80 (1% v/v) exhibited its own antimicrobial effect, which may have influenced the results obtained, as the observed activity likely reflects a synergistic interaction between the oil and the surfactant.

## 5 Conclusion

Both TTO and CHX mouthwashes significantly reduced plaque accumulation, gingival inflammation, and bacterial load after 15 days of use. While CHX demonstrated stronger antibacterial activity, TTO achieved comparable clinical outcomes with fewer adverse effects. These findings will serve as foundation for future larger trials to confirm TTO as a safe and effective adjunct for managing plaque-induced gingivitis. Further studies with larger samples and longer follow-up are warranted to confirm these results and optimize formulation safety, particularly by ensuring the use of non-toxic and biologically inert emulsifiers to avoid potential antimicrobial bias.

## Data Availability

The raw data supporting the conclusions of this article will be made available by the authors, without undue reservation.

## Supplementary material

SUPPLEMENTARY TABLE S1 Sociodemographic and behavioural characteristics of the study sample

## References

[1] Chapple ILC Mealey BL Van Dyke TE Bartold PM Dommisch H Eickholz P. Periodontal health and gingival diseases and conditions on an intact and a reduced periodontium: consensus report of workgroup 1 of the 2017 world workshop on the classification of periodontal and peri-implant diseases and conditions. J Periodontol. (2018) 89(S1). 10.1002/JPER.17-0719 29926944

[2] Caton JG Armitage G Berglundh T Chapple ILC Jepsen S Kornman KS. A new classification scheme for periodontal and peri-implant diseases and conditions – Introduction and key changes from the 1999 classification. J Periodontol. (2018) 89(S1). 10.1002/JPER.18-0157 29926946

[3] Gare J Kanoute A Orsini G Gonçalves LS Ali Alshehri F Bourgeois D. Prevalence, severity of extension, and risk factors of gingivitis in a 3-month pregnant population: a multicenter cross-sectional study. J Clin Med. (2023) 12(9):3349. 10.3390/jcm12093349 37176789 PMC10179599

[4] Relvas M López-Jarana P Monteiro L Pacheco JJ Braga AC Salazar F. Study of prevalence, severity and risk factors of periodontal disease in a Portuguese population. J Clin Med. (2022) 11(13):3728. 10.3390/jcm11133728 35807013 PMC9267442

[5] Perić M Marhl U Gennai S Marruganti C Graziani F. Treatment of gingivitis is associated with reduction of systemic inflammation and improvement of oral health-related quality of life: a randomized clinical trial. J Clin Periodontol. (2022) 49(9):899–910. 10.1111/jcpe.13690 35762095

[6] Chapple ILC Van Der Weijden F Doerfer C Herrera D Shapira L Polak D. Primary prevention of periodontitis: managing gingivitis. J Clin Periodontol. (2015) 42(S16). 10.1111/jcpe.12366 25639826

[7] Akram Z Shafqat S Aati S Kujan O Fawzy A. Clinical efficacy of probiotics in the treatment of gingivitis: a systematic review and meta-analysis. Aust Dent J. (2020) 65(1):12–20. 10.1111/adj.12733 31682012

[8] Reiniger APP Maier J Wikesjö UME Moreira CHC Kantorski KZ. Correlation between dental plaque accumulation and gingival health in periodontal maintenance patients using short or extended personal oral hygiene intervals. J Clin Periodontol. (2021) 48(6):834–42. 10.1111/jcpe.13448 33751652

[9] Preethanath RS Ibraheem WI Anil A. Pathogenesis of gingivitis. In: Sridharan G Sukumaran A Eddin Omar Al Ostwani A, editors. Oral Diseases. IntechOpen (2020).

[10] Brun A Petit C Huck O Bouchard P Carra MC Gosset M. La parodontite : un risque sous-estimé des maladies cardiovasculaires. Médecine/Sciences. (2024) 40(1):35–41. 10.1051/medsci/2023193 38299901

[11] Cichońska D Mazuś M Kusiak A. Recent aspects of periodontitis and Alzheimer’s disease—a narrative review. Int J Mol Sci. (2024) 25(5):2612. 10.3390/ijms25052612 38473858 PMC10931712

[12] Clarke JK. On the bacterial factor in the aetiology of dental caries. Br J Exp Pathol. (1924) 5(3):141–7.

[13] Lin Y Chen J Zhou X Li Y. Inhibition of Streptococcus mutans biofilm formation by strategies targeting the metabolism of exopolysaccharides. Crit Rev Microbiol. (2021) 47(5):667–77. 10.1080/1040841X.2021.1915959 33938347

[14] Kamath NP Tandon S Nayak R Naidu S Anand PS Kamath YS. The effect of aloe vera and tea tree oil mouthwashes on the oral health of school children. Eur Arch Paediatr Dent. (2020) 21(1):61–6. 10.1007/s40368-019-00445-5 31111439

[15] Lakshmi SSJ Leela KV. A review on updated Species list of Viridans streptococci causing infective endocarditis. J Pure Appl Microbiol. (2022) 16(3):1590–4. 10.22207/JPAM.16.3.26

[16] Fang Y Chen X Chu CH Yu OY He J Li M. Roles of Streptococcus mutans in human health: beyond dental caries. Front Microbiol. (2024) 15:1503657. 10.3389/fmicb.2024.1503657 39749137 PMC11693680

[17] Khalil A Mostafa M El-Araby S El-Bouseary M. Evaluation of the effect of tea tree oil mouthwash on Streptococcus Mutans as compared with chlorohexidine in A group of Egyptian children. Al-Azhar J Dent. (2023) 10(1). 10.58675/2974-4164.1476

[18] Dutt P Kr Rathore P Khurana D. Chlorhexidine - an antiseptic in periodontics. IOSR J Dent Med Sci. (2014) 13(9):85–8. 10.9790/0853-13968588

[19] Poppolo Deus F Ouanounou A. Chlorhexidine in dentistry: pharmacology, uses, and adverse effects. Int Dent J. (2022) 72(3):269–77. 10.1016/j.identj.2022.01.005 35287956 PMC9275362

[20] Moreno ALDM De Moraes Melo Neto CL Coelho Goiato M Moreno NVDA Dos Santos DM De Lima CC. Effect of chlorhexidine and tea tree oil on reducing the number of oral microorganisms. Eur J Dent. (2024) 18(01):397–400. 10.1055/s-0043-1769900 37532117 PMC10959613

[21] Wasey F Tantray S Ahluwalia R Khan MS. Comparative evaluation of 0.25% lemongrass oil mouthwash and 0.2% chlorhexidine mouthwash in fixed orthodontic patients suffering from gingivitis. J Contemp Dent Pract. (2023) 24(6):396–402. 10.5005/jp-journals-10024-3516 37534506

[22] Thangavelu A Kaspar S Kathirvelu R Srinivasan B Srinivasan S Sundram R. Chlorhexidine: an elixir for periodontics. J Pharm Bioallied Sci. (2020) 12(5):57. 10.4103/jpbs.JPBS_162_20 33149431 PMC7595540

[23] Viganò L Nosotti MG Orlova N Casu C. Use of chlorhexidine, side effects and antibiotic resistance. Available online at: https://www.researchgate.net/publication/325811996_Use_of_chlorhexidine_side_effects_and_antibiotic_resistance

[24] Abbood HM Hijazi K Gould IM. Chlorhexidine resistance or cross-resistance, that is the question. Antibiotics. (2023) 12(5):798. 10.3390/antibiotics12050798 37237701 PMC10215778

[25] Penfold AR Morrison FR. Some Notes on the Essential oil of Melaleuca alternifolia (1937).

[26] Yang SY Lee SH Park OB An HR Yu YH Hong EB. Antibacterial effects of tea tree oil and mastic oil to Streptococcus mutans. J Dent Hyg Sci. (2023) 23(1):51–9. 10.17135/jdhs.2023.23.1.51

[27] Iacovelli F Romeo A Lattanzio P Ammendola S Battistoni A La Frazia S. Deciphering the broad antimicrobial activity of Melaleuca alternifolia tea tree oil by combining experimental and computational investigations. Int J Mol Sci. (2023) 24(15):12432. 10.3390/ijms241512432 37569803 PMC10420022

[28] Song YM Zhou HY Wu Y Wang J Liu Q Mei YF. *In vitro* evaluation of the antibacterial properties of tea tree oil on planktonic and biofilm-forming Streptococcus mutans. AAPS PharmSciTech. (2020) 21(6):227. 10.1208/s12249-020-01753-6 32767025

[29] Brun P Bernabè G Filippini R Piovan A. *In Vitro* antimicrobial activities of commercially available tea tree (Melaleuca alternifolia) essential oils. Curr Microbiol. (2019) 76(1):108–16. 10.1007/s00284-018-1594-x 30421144

[30] Karbach J Ebenezer S Warnke P Behrens E Al-Nawas B. Antimicrobial effect of Australian antibacterial essential oils as alternative to common antiseptic solutions against clinically relevant oral pathogens. Clin Lab. (2015). 10.7754/Clin.Lab.2014.140714 25807639

[31] Patri G. Role of herbal agents - tea tree oil and Aloe vera as cavity disinfectant adjuncts in minimally invasive dentistry- an *in vivo* comparative study. J Clin Diagn Res. (2017). 10.7860/JCDR/2017/27598.10147 PMC5583794 28892888

[32] Bugarcic A Bowles EJ Summer K Agnew T Barkla B Lauche R. Australian tea tree (Melaleuca alternifolia) oil: an updated review of antimicrobial and other medicinal properties. Phytomedicine Plus. (2025) 5(3):100846. 10.1016/j.phyplu.2025.100846

[33] Liu Y Tang X Zhang H Zheng L Lai P Guo C. Terpinen-4-ol improves lipopolysaccharide-induced macrophage inflammation by regulating glutamine metabolism. Foods. (2024) 13(12):1842. 10.3390/foods13121842 38928786 PMC11202924

[34] Bekhof AMW Van Hunsel FPAM Van De Koppel S Woerdenbag HJ. Safety assessment and adverse drug reaction reporting of tea tree oil (Melaleuca aetheroleum). Phytother Res. (2023) 37(4):1309–18. 10.1002/ptr.7687 36420525

[35] Kfoury M Fourmentin S. Les huiles essentielles : renaissance d’ingrédients naturels et durables. Technol Innov. (2024) 9(1).

[36] Tea Tree Oil Uses, Benefits & Dosage. Available online at: Drugs.com, https://www.drugs.com/npp/tea-tree-oil.html [Accessed October 17, 2025]

[37] Salvatori C. A comparative study of antibacterial and anti-inflammatory effects of mouthrinse containing tea tree oil. Oral Implantol. (2017) 10(1):59. 10.11138/orl/2017.10.1.059 PMC5516420 28757937

[38] Iseppi R Mariani M Condò C Sabia C Messi P. Essential oils: a natural weapon against antibiotic-resistant Bacteria responsible for nosocomial infections. Antibiotics. (2021) 10(4):417. 10.3390/antibiotics10040417 33920237 PMC8070240

[39] Rahbari S Rajabi M Pournasiri Z. The comparative efficacy of tea tree oil body wash versus chlorhexidine body wash to prevent colonization with methicillin-resistant staphylo-coccus Aureus in a pediatric unit. J Pharm Care. (2023). 10.18502/jpc.v11i2.13362

[40] Zhang C Liu B Hu J Zhao L Zhao H. The effect of local application of tea tree oil adjunctive to daily oral maintenance and nonsurgical periodontal treatment: a systematic review and meta-analysis of randomised controlled studies. Oral Health Prev Dent. (2024) 22(1):211–22. 38864380 10.3290/j.ohpd.b5458585 PMC11619907

[41] Ramadan MA Shawkey AE Rabeh MA Abdellatif AO. Expression of P53, BAX, and BCL-2 in human malignant melanoma and squamous cell carcinoma cells after tea tree oil treatment *in vitro*. Cytotechnology. (2019) 71(1):461–73. 10.1007/s10616-018-0287-4 30599074 PMC6368524

[42] Casalle N de Andrade CR. Cytotoxic and mutagenic capacity of TTO and terpinen-4-ol in oral squamous cell carcinoma. bioRxiv 2020.01.03.893735. 10.1101/2020.01.03.893735

[43] De Assis KMA Rêgo DAR De Melo DF Da Silva LM Oshiro- Júnior JA Formiga FR. Therapeutic potential of Melaleuca alternifolia essential oil in new drug delivery systems. Curr Pharm Des. (2020) 26(33):4048–55. 10.2174/1381612826666200305124041 32133957

[44] Hopewell S Chan AW Collins GS Hróbjartsson A Moher D Schulz KF. CONSORT 2025 statement: updated guideline for reporting randomised trials. Br Med J:2025:e081123. 10.1136/bmj-2024-081123 PMC11995449 40228833

[45] ISO 10993-5:2009. Évaluation Biologique des Dispositifs Médicaux Partie 5: Essais Concernant la Cytotoxicité in Vitro (2009).

[46] Rajkowska K Nowak A Kunicka-Styczyńska A Siadura A. Biological effects of various chemically characterized essential oils: investigation of the mode of action against Candida albicans and HeLa cells. RSC Adv. (2016) 6(99):97199–207. 10.1039/C6RA21108A

[47] Giannelli M Chellini F Margheri M Tonelli P Tani A. Effect of chlorhexidine digluconate on different cell types: a molecular and ultrastructural investigation. Toxicol in Vitro. (2008) 22(2):308–17. 10.1016/j.tiv.2007.09.012 17981006

[48] Faria G Celes MRN De Rossi A Silva LAB Silva JS Rossi MA. Evaluation of chlorhexidine toxicity injected in the paw of mice and added to cultured L929 fibroblasts. J Endod. (2007) 33(6):715–22. 10.1016/j.joen.2006.12.023 17509413

[49] De Oliveira MS Da Silva NP De Paula MSA Porto DL Aragão CFS Segura MEC. Poly(*ε*-caprolactone) nanocapsules coated with chitosan optimize the antimicrobial activity of tea tree oil and azithromycin against oral pathogens. Microb Pathog. (2025) 206:107820. 10.1016/j.micpath.2025.107820 40543634

[50] Singh N Puzhankara L Kedlaya MN Ramanarayanan V. Effectiveness of tea tree oil versus chlorhexidine in the treatment of periodontal diseases: a systematic review. Evid Based Dent. (2022). 10.1038/s41432-022-0259-6 35821403

[51] Mahapatra A Panda S Tumedei M Panda S Das AC Kumar M. Clinical and microbiological evaluation of 0.2% tea tree oil mouthwash in prevention of dental biofilm-induced gingivitis. Dent J. (2025) 13(4):149. 10.3390/dj13040149 PMC12025935 40277479

[52] Reddy DV Bennadi DD. Effectiveness of tea tree oil and chlorhexidine as mouth rinse in the control of dental plaque and chronic gingivitis – A comparative study. Clin Med. (2020) 7(08).

[53] Wara-aswapati N Krongnawakul D Jiraviboon D Adulyanon S Karimbux N Pitiphat W. The effect of a new toothpaste containing potassium nitrate and triclosan on gingival health, plaque formation and dentine hypersensitivity. J Clin Periodontol. (2005) 32(1):53–8. 10.1111/j.1600-051X.2004.00631.x 15642059

